# Conservation in Mammals of Genes Associated with Aggression-Related Behavioral Phenotypes in Honey Bees

**DOI:** 10.1371/journal.pcbi.1004921

**Published:** 2016-06-30

**Authors:** Hui Liu, Gene E. Robinson, Eric Jakobsson

**Affiliations:** 1 Center for Biophysics and Computational Biology, University of Illinois at Urbana-Champaign, Urbana, Illinois, United States of America; 2 Carl R. Woese Institute for Genomic Biology, University of Illinois at Urbana-Champaign, Urbana, Illinois, United States of America; 3 Department of Entomology, University of Illinois at Urbana-Champaign, Urbana, Illinois, United States of America; 4 Neuroscience Program, University of Illinois at Urbana-Champaign, Urbana, Illinois, United States of America; 5 Beckman Institute for Advanced Science and Technology, University of Illinois at Urbana-Champaign, Urbana, Illinois, United States of America; 6 National Center for Supercomputing Applications, University of Illinois at Urbana-Champaign, Urbana, Illinois, United States of America; 7 Department of Molecular and Integrative Physiology, University of Illinois at Urbana-Champaign, Urbana, Illinois, United States of America; The University of Texas, UNITED STATES

## Abstract

The emerging field of sociogenomics explores the relations between social behavior and genome structure and function. An important question is the extent to which associations between social behavior and gene expression are conserved among the Metazoa. Prior experimental work in an invertebrate model of social behavior, the honey bee, revealed distinct brain gene expression patterns in African and European honey bees, and within European honey bees with different behavioral phenotypes. The present work is a computational study of these previous findings in which we analyze, by orthology determination, the extent to which genes that are socially regulated in honey bees are conserved across the Metazoa. We found that the differentially expressed gene sets associated with alarm pheromone response, the difference between old and young bees, and the colony influence on soldier bees, are enriched in widely conserved genes, indicating that these differences have genomic bases shared with many other metazoans. By contrast, the sets of differentially expressed genes associated with the differences between African and European forager and guard bees are depleted in widely conserved genes, indicating that the genomic basis for this social behavior is relatively specific to honey bees. For the alarm pheromone response gene set, we found a particularly high degree of conservation with mammals, even though the alarm pheromone itself is bee-specific. Gene Ontology identification of human orthologs to the strongly conserved honey bee genes associated with the alarm pheromone response shows overrepresentation of protein metabolism, regulation of protein complex formation, and protein folding, perhaps associated with remodeling of critical neural circuits in response to alarm pheromone. We hypothesize that such remodeling may be an adaptation of social animals to process and respond appropriately to the complex patterns of conspecific communication essential for social organization.

## Author Summary

Sociogenomics explores the relationship between social behavior and the genome. An important issue is the extent to which results from social insects can be used to understand social behavior in other animals. We address this question through computational studies of previously published experimental data on patterns of brain gene expression in honey bees in response to particular environmental conditions and stimuli. We found that for one particular stimulus, response to alarm pheromone, the set of honey bee genes differentially expressed in the brain contains disproportionately large numbers of genes also found in mammals, including humans. This enrichment in orthologous genes suggests surprisingly strong similarities in socially responsive genetic circuits common to honey bees and mammals. A large number of the human counterparts of these genes are important for regulating protein folding, protein metabolism, and regulation of protein complex formation, perhaps reflecting changes in macromolecular complexes involved in remodeling critical neural circuits in response to the alarm pheromone. Noting that alarm pheromone is a component of the honey bee’s communication system, we hypothesize that such rapid remodeling may be an adaptation in the brain cells of social animals to deal with the complex patterns of conspecific signaling essential for social organization.

## Introduction

Social behavior, like phenotypes of any level of complexity, is regulated by the activity of genomic networks and resulting gene expression. At the same time that specific examples of genes influencing behavior were being discovered empirically[1,2], the field of systems biology was developing[3]. The essence of systems biology is to use computation and genomic technologies to enable detailed observation at the sequence level of the dynamics of cell, tissue, and organism responses to specific challenges. The power of systems biology is that it enables comprehensive dynamic patterns of transcription, translation, post-translational modification, and functioning of gene products to be observed and analyzed. These approaches provide fertile ground for the development of testable hypotheses and ultimately confident inferences about the relationship between the genome and phenome (the sum total of the organism’s phenotypic traits), even when the phenome is based on complex patterns of gene interactions. The systems approach has catalyzed the development of the fields of evo-devo[4] and, more recently, sociogenomics [1]. Sociogenomics focuses on how genes influence social behavior [2] and how environmental attributes—especially those related to the social environment—influence genome activity [5].

Evo-devo has led to new insights into the molecular basis for the evolution of morphological novelties, molecular mechanisms underlying the development of morphology in the individual, and how development responds to the environment on a genomic level. Specifically, it has shown that the major (but not only) driver in evolution of form has been changes in expression patterns of functionally conserved genes [6] Synergistically, sociogenomics seeks to provide insights into the evolution of social behavior, the genomic mechanisms underlying social behavior in an individual and a species, and how social behavior is influenced by the environment at the genomic level [1]. Similar to the evolution of biological form, the evolution of a vertebrate social decision-making network has been shown to be largely (but again not entirely) by variations in conserved genes and networks [7].

One approach to sociogenomics is hypothesis-driven. In this approach, researchers begin with a hypothesis about the role of a gene or a group of genes in social behavior based on prior knowledge of the function or activity of those genes. As an example of this approach, O’Tuathaigh et al [8] observed that the knockout of the mouse ortholog of the human schizophrenia gene *neuregulin 1* disrupted social novelty behavior, but left spatial learning and working memory processes intact. This gene has close homologs throughout the vertebrates, putative orthologs in arthropods, and significantly similar homologs annotated as coding for cell wall anchoring proteins in some bacteria.

By contrast, systems biology studies often begin with no hypothesis (except the fundamental one that social behavior has genomic bases) and scan comprehensively to see what correlations emerge. As an example of this approach, Cummings et al [9] identified differential gene expression patterns in the response of female swordtail fish to different classes of conspecifics (attractive males, unattractive males, other females). This broad systems approach was extended across multiple species in a study in which molecular orthology and comparative brain morphology were used to identify social behavior networks in vertebrates [10]. This work nicely illustrates the above-mentioned convergence of sociogenomics and evo-devo.

The studies cited above highlight the fact that understanding the genomic correlates of human social behavior requires us to use a variety of model organisms, in part because of the invasive nature of many experimental protocols. Ebstein et al [2] observed that “Human beings are an incredibly social species and along with eusocial insects engage in the largest cooperative living groups in the planet’s history.” This leads to the question: to what extent are there relevant genomic correlates between eusocial insects and humans, given that the last common ancestor of eusocial insects and humans lived approximately 670 million years ago [11] and almost certainly was very different in appearance from either an insect or a vertebrate. It may be that both eusocial insect and human social traits are elaborations and modifications of underlying patterns that were present in a common ancestor, even if the elaborations occurred independently[12]. As a corollary to this view, some species in the lineages leading to both insects and chordates would have lost or inhibited expression of these traits, while other species such as the eusocial insects and humans would have continued to express them and use them as a set of building blocks for social behavior. To the extent this is true, comparative genomics of eusocial insect social behavior and human social behavior may yield insights into some of the most fundamental aspects of the genomics of social behavior. This would be an example of the general principle that conserved elements between species separated by great evolutionary distance are likely to be universal building blocks of common aspects of the species’ phenomes [6].

Among the eusocial insects, the honey bee is a valuable model organism. Many experiments have linked brain gene expression patterns to social behavioral characteristics and environmental stimuli, and the honey bee genome has been sequenced [13]. In addition, individual members of a honey bee colony have well-defined social roles. It is known that the division of labor within the hive is based on both genetic differences between individual honey bees and also on environmental influences that include visual, tactile, and chemical signals that colony members send to each other, as well as environmental influences external to the colony [13]. However, the interplay between these factors is poorly defined with respect to variation in particular genes or regulatory domains in the genome. There are statistically detectable hereditary tendencies for particular honey bees to play particular social roles, but the individual bee’s social role is determined by the interactions between both social and environmental factors, as well as heredity. Understanding this complex interplay of internal and external factors is central to sociogenomics.

One way to make a connection between honey bee and human sociogenomics is by inference of genetic orthology. Unfortunately, orthology is of necessity not verifiable in the same fashion as other techniques of bioinformatics, since it involves theoretical reconstruction of an evolutionary history that cannot be experimentally replicated. Thus, there is no reliable validation set on which to test a method. Different reasonable ways of creating orthologies may give significantly different results [14]. Whether one makes a liberal or conservative interpretation of orthological relationships produced by a particular method depends on the context, in particular whether one is concerned about contamination by false positive identifications of orthologs, or more concerned about loss of information by false negatives. In the present paper, we use a new application of orthology to test the hypothesis that the social behavior of honey bees and other metazoans, including humans, has common fundamental genomic building blocks.

This paper seeks to explore the degree of relevant sequence conservation between honey bees and humans. Our starting point is the data set from Alaux et al [15], who used microarrays to analyze differential brain gene expression patterns exhibited by individual honey bees of different genetic backgrounds, engaged in different social roles and in different colony environments. African and European honey bees are subspecies of the Western honey bee, *Apis mellifera*, and they differ from each other in their hive defense behavior in a number of ways that have been summarized as a social behavioral counterpart to variations of threshold and intensity of the “flight or fight” response seen in vertebrate organisms; African bees are much more aggressive than European bees [16]. In general, different phenotypes may arise from either differences in gene function or from different patterns of gene expression [17]. In the African and European honey bees it is presumed that the different phenotypes are largely the result of different patterns of gene expression, and differences in the expression of hundreds of genes in the brain have been reported [15]. Bees in Alaux et al were raised in a cross-fostered experimental design to examine the influences of genetic background and social environment on brain gene expression.

We analyzed the above-cited [15] data sets to explore the following two questions: 1) to what extent are the differentially expressed genes associated with social behavior in the honey bee conserved across the Metazoa; and 2) through analysis of the highly conserved genes, is it possible to infer that there are likely to be gene co-expression patterns associated with social behavior that are common to a wide range of metazoans, including humans?

## Results

We examined eight sets of social behavior-related differentially expressed genes from Alaux et al [15]. They are described in Table 1 and S3 Table.

**Table 1:** Summary of the sets of differentially expressed genes analyzed in this study.

Set Number and Name	Number of genes	Number of genes mapable	Set Description
1. Alarm_Pheromone (large behavioral phenotype difference)	344	275	European bees exposed to alarm pheromone vs European control bees
2. Old_vs_Young (large behavioral phenotype difference)	1125	899	European old bees vs European young bees
3. Soldier_CG (large behavioral phenotype difference)	664	512	African colony soldier bees vs European colony soldier bees
4. Soldier_WG (large behavioral phenotype difference)	396	308	Genetically African soldier bees vs genetically European soldier bees
5. Forager_CG (smaller behavioral phenotype difference)	236	180	African colony forager bees vs European colony forager bees
6. Forager_WG (smaller behavioral phenotype difference)	41	22	Genetically African forager bees vs genetically European forager bees
7. Guard_CG (smaller behavioral phenotype difference)	336	248	African colony guard bees vs European colony guard bees
8. Guard_WG (smaller behavioral phenotype difference)	173	132	Genetically African guard bees vs genetically European guard bees

“Number of genes mapable” are the number of differentially expressed genes whose IDs are mapable to InParanoid ortholog database. All eight sets in this table are from [15]. For each social class of bee (forager, guard, soldier) there are four subpopulations: AE (Genetically African bees in European colony), AA (Genetically African bees in African colony), EA (Genetically European bees in African colony), EE (Genetically European bees in European colony). For the sets labeled “WG” (Worker Genotype) AE and AA are integrated via ANOVA statistics into one set and compared to the integrated set comprised of EE and EA. For the sets labeled “CG” (Colony Genotype) AA and EA are integrated via ANOVA into one set and compared to the integrated set comprised of AE and EE. Sets 1, 2, 3, 4 are associated with very large behavioral differences in aggression during hive defense. Sets 5, 6, 7, 8 are sets associated with smaller behavioral differences.

**Fig 1:**
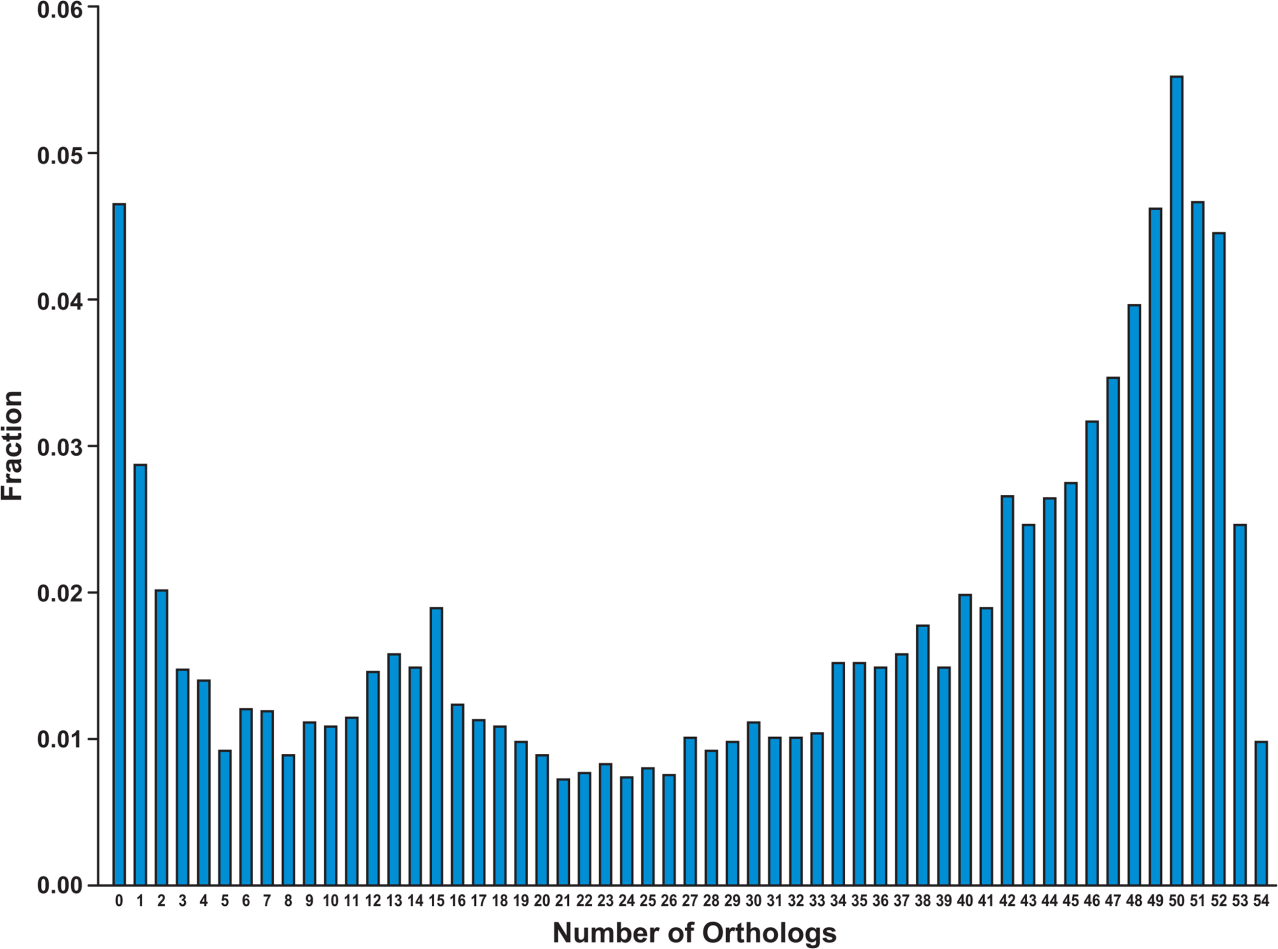
Normalized distribution of the number of all of the “Array-Spotted” Honey Bee genes” orthologs in 54 species. “Array-Spotted” means that these genes are present in the InParanoid database and spotted on the Honey Bee Oligonucleotide Microarray. There are 7462 such honey bee genes. X-axis is the number of orthologs in 54 species (53 metazoan species+ yeast). Y-axis is the percentage of these 7462 honey bee genes that have the corresponding number of orthologs. The species are identified in the label of the cladogram at the bottom of Fig 3A. The three relative maxima in the distribution corresponding to 0, 15, and 50 orthologs correspond respectively to genes unique to honeybees, genes conserved among arthropods, and genes widely conserved across metazoan.

**Fig 2:**
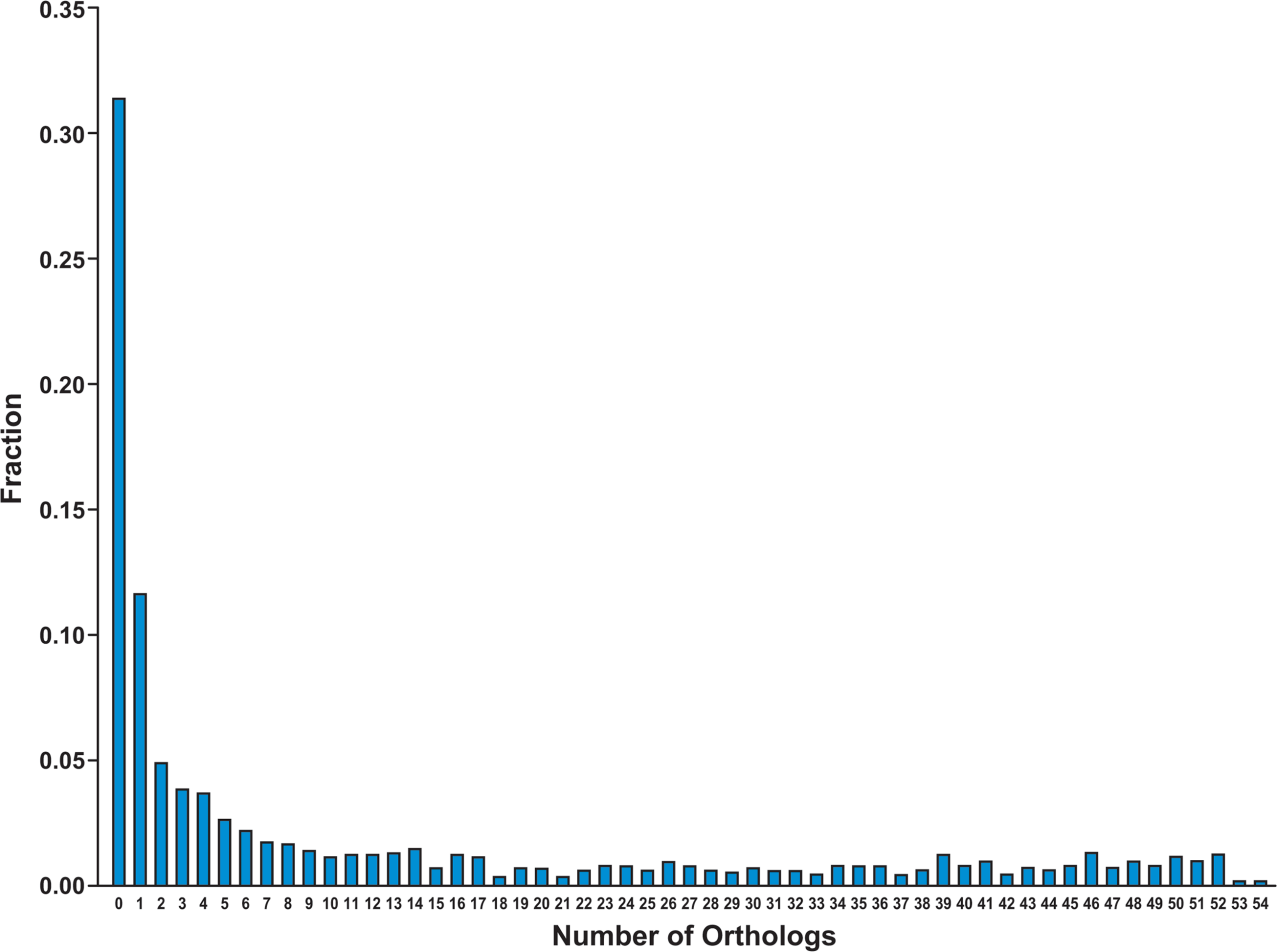
Normalized distribution of the number of all of the “Array-Unspotted” Honey Bee genes” orthologs in 54 species. “Array-Unspotted” means that these genes are present in the InParanoid database but not spotted on the Honey Bee Oligonucleotide Microarray. There are 1631 such honey bee genes. X-axis is the number of orthologs in 54 species (53 metazoan species+ yeast). Y-axis is the percentage of these 1631 honey bee genes that have the corresponding number of orthologs. (Note vertical scale difference between Figs 1 and 2).

For each of the honey bee genes on the microarray, we interrogated the InParanoid database of orthologous genes [18] to ascertain how many orthologs each gene had within a set of organisms including yeast plus 53 metazoa. The results are plotted in Fig 1 in the form of a histogram that shows what fraction of the genes had 1, 2, …. 54 orthologs. Just under 5% of the genes had no orthologs in the InParanoid set; within this data set they were unique to the honey bee. Of the 54 species we compared to the honey bee, 17 were insects. The position of the first peak in the distribution (at 15 orthologs) is due to genes that were largely conserved in insects and were uncommon in other metazoan lineages. The position of the second peak (at 50 orthologs) was due to genes that are broadly conserved across the metazoa. There were 1631 honey bee genes in the InParanoid database that were not included on the microarray. Approximately one third of those 1631 genes not included were unique to the honey bee (Fig 2). Over 50% of the 1631 genes had fewer than four orthologs in the set of 54 species analyzed. This relative lack of conservation of the excluded genes is largely a function of how the microarray was designed [15] Since the major conclusions of this paper were based on orthology to other metazoa, and since the genes excluded from the analysis had relatively few such orthologs, the conclusions are unlikely to be significantly affected by the exclusion of these genes.

### Ortholog Distribution across the Metazoa of Honey Bee Genes Related to Behavioral Phenotypes

Table 2 provides the overall summary of the results. At the 0.05 significance level (based on Benjamini-corrected p-values), three of the sets were selectively enriched in genes conserved across the Metazoa: the Alarm_Pheromone set, the Old_vs_Young set, and the Soldier_CG (colony genotype) set. By the same standard of significance, the Guard_CG, Guard_WG (worker genotype), and the Forager_WG sets were significantly depleted in highly conserved genes (i.e., the Benjamini-corrected p-value was over 0.95).

**Table 2:** Statistics of the ortholog count data of sets of differentially expressed honey bee genes.

Set Name	Total Number of Orthologs	P value	Set Size	Average Number of Orthologs per gene	Standard Deviation
'Alarm_Pheromone'	9402	0.011539*	275	34.19	16.99
'Old_vs_Young'	29722	0.011921*	899	33.06	17.61
'Soldier_CG'	17062	0.022775*	512	33.32	16.65
'Soldier_WG'	9461	0.86155	308	30.72	18.43
'Forager_CG'	5403	0.91136	180	30.02	17.75
'Forager_WG'	487	0.99324	22	22.14	18.48
'Guard_CG'	7098	0.99716	248	28.62	17.63
'Guard_WG'	3529	0.99936	132	26.73	17.67

Names of sets of differentially expressed genes are the same as tabulated in Table 1. The p-values for the mean number of orthologs are calculated by random sampling, see statistics part of Methods.

The p-values for the three sets selectively enriched in genes conserved across the metazoan are bolded and marked by asterisk.

**Fig 3:**
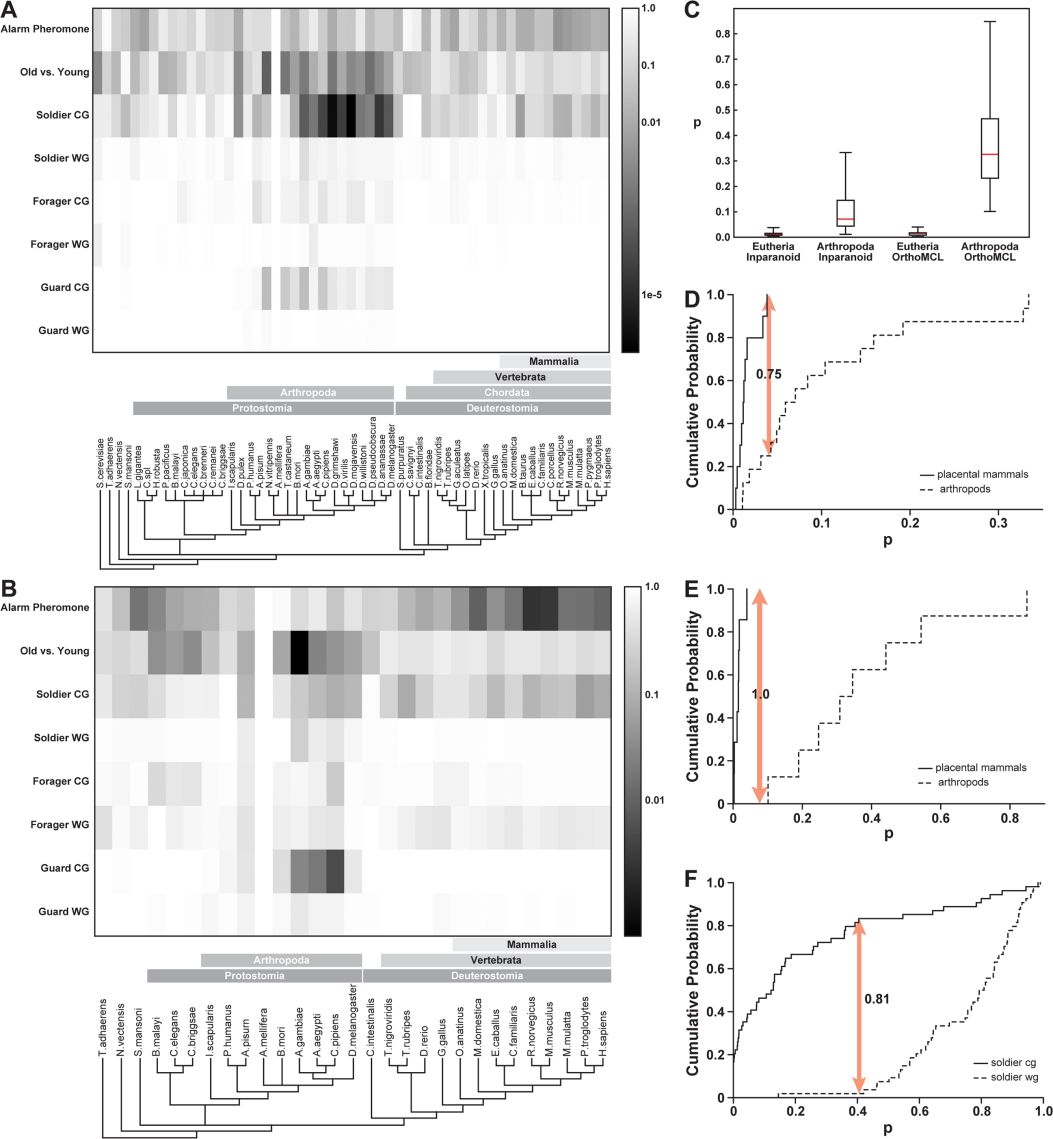
Heat maps and statistical analysis of the p-values of number of orthologs for each species and each set. Each row of the heat maps in Figs 3a and 3b represents one of the honey bee experimental sets. Each column represents one species. Fig 3a is based on the InParanoid ortholog database and 3 b is based on the OrthoMCL database. The white represents the honey bee. The species are ordered along the x-axis by evolutionary distance from the honey bee based on NCBI taxonomy common tree (http://www.ncbi.nlm.nih.gov/Taxonomy/CommonTree/wwwcmt.cgi). The order is further refined based on the tree from Flybase (http://flybase.org/blast/species_tree.png), WormBook [42] and UCSC Genome Browser [43]. The evolutionary relationships are illustrated by the cladogram along the base of each heat map. Those species to the left of the honey bee (the white vertical column) belong to lineages that diverged from the insects earlier than the insects diverged from the lineage leading to the mammals. The species immediately to the right of the honey bee are insects and other arthropods. The species at the far right are mammals. Between the insects and the mammals are marine invertebrates, marine chordates, fish, amphibians, and birds. The shading code (vertical bar on the right hand side of the figure) represents the p-value for statistical significance of enrichment of orthology in each species relative to the degree of orthology of all the genes on the microarray. The numerical data for these plots are in S1 Table. Panel c represents the enhanced orthology between the honey bee alarm pheromone set and the placental mammals by means of boxplots. Reading from left to right the boxplots show: i) the distribution of individual species p-values for orthology enhancement between the honey bees and the placental mammals using the InParanoid database, ii) the distribution of individual species p-values for orthology enhancement between the honey bees and their fellow arthropods using the InParanoid data base, iii) the same as i) but using the OrthoMCL database, and iv) the same as ii) but using the OrthoMCL database. It is seen that the p-values for orthology enhancement of the honey bee alarm pheromone set are much much lower (and therefore more favorable) for placental mammals than for arthropods. This is in spite of the fact that overall the honey bee is much more closely related to the other arthropods than to the mammals, as indicated by cladograms in Fig 3a and 3b. The relative positions of the boxplots in Fig 3c are just the opposite of what would pertain if the degree of orthology followed the species relationship. Fig 3d shows the Kolmogorov-Smirnov cumulative fraction difference plot for the distributions of p-values for orthology enrichment of the Alarm Pheromone gene set against the placental mammals (solid trace) and against the arthropods, using the InParanoid orthology database. The horizontal axis is p-values. Each vertical position on each trace is the fraction of p-values making up that trace whose p-value is below the indicated value. One important feature of such a plot is the maximum vertical distance between the two traces, D. In this figure, D is 0.75. P, the likelihood that this difference would be achieved by chance for a distribution with this number of data points, is 0.001 Fig 3e shows the same graph as Fig 3d using the OrthoMCL orthology database. In this graph the value of D is 1.0, since there is no overlap between the distributions at all. P, the likelihood that this degree of separation would occur by chance among members of the same underlying distribution, is vanishingly small. Based on the Kolmogorov-Smirnov statistics, depicted in Fig 3d and 3e we can say with confidence that the Alarm Pheromone honey bee gene set is relatively more enriched in orthologs to placental mammals than in orthologs to other arthropods. Fig 3f shows the Kolmogrov-Smirnov cumulative fraction plot for the differences between the p-values for orthology enrichment for the soldier cg and soldier wg sets. It is seen that the degree of conservation is dramatically higher for the cg than the wg set, which can also be inferred qualitatively from the shading in the heat map of Fig 3a. The value of D, the greatest vertical distance between the two traces shown by the double-headed arrow, is 0.81. P, the probability that the difference between the two traces is due to chance, is < .0005.

We examined the conservation pattern with each of the species used in the analysis, via a heat map, for the eight data sets (Fig 3). These analyses were based on the InParanoid orthology database (Fig 3A) and the OrthoMCL orthology database, which contained a smaller number of species (Fig 3B). A relatively high degree of conservation was distributed across a wide range of metazoans for Old_vs_Young, Alarm_Pheromone, and Soldier_CG sets. For Soldier_CG and Old_vs_Young, the most significant conservation was within the insect group. For the Alarm_Pheromone set, on the other hand, Fig 3A and 3B indicate that the greatest degree of conservation was in mammals. Another way of visualizing the greater degree of conservation in mammals is in Fig 3C, which shows box-and-whisker plots of the distribution of p-values for orthology enrichment of the honeybee Alarm Pheromone set for the honeybee’s closest relatives (arthropods) and for human’s closest relatives, the highly social placental mammals. For both the InParanoid and the OrthoMCL databases, the degree of conservation clearly tends higher (lower p-value) for the mammals than for the arthropods. To test the statistical significance of the greater conservation of the Alarm Pheromone set in placental mammals we applied the Kolgomorov-Smirnov (KS) test, which is a standard method for assessing the significance of the difference between two unbinned distributions [19]. Fig 3D and 3E show the KS comparison cumulative fraction plots for the arthropod/placental mammal p-value distributions from the Alarm Pheromone gene set using the InParanoid and the OrthoMCL orthology databases, respectively. In these plots the horizontal axis represents the range of p-values for orthology enrichment and the vertical axis represents the fraction of species in each class whose p-values are below a particular level. The critical features of each graph are the quantity D, representing the maximum different between the plots for the two distributions, and a corresponding P (The likelihood that the difference between the distributions arose by chance, which is a function of D and of the number of values in the two distributions; see Press et al, 1992 [19], for exact expression for computing P). For the InParanoid set, the value of D is 0.75, meaning that the lowest quartile of the p-values for the arthropods is within the range of the p-values for the placental mammals, while the upper 75% of the arthropod p-values is larger than any of the placental mammals. The value of P (the likelihood that this discrepancy between the distributions arose by chance) is .001. In Fig 3E, which shows the comparison cumulative fraction plots for the distributions as derived from the OrthoMCL data base, the value of D is 1, because there is no overlap between the distributions. The largest p-value of any of the placental mammals is smaller than the smallest p-value for any of the arthropods. Therefore the value of P is vanishingly small. Based on these statistics, we confidently conclude that the genes differentially expressed in the honey bee in response to the alarm pheromone are systematically enriched in orthologs to genes in placental mammals.

This finding suggests that, of all the gene sets analyzed, the set differentially expressed in response to the alarm pheromone stimulus was most likely to include genes from genomic networks common to honey bees and mammals. The analyzed gene expression data and the results of the orthology searches are provided in spreadsheet form in S3 Table.

In order to be conservative in our assignment of orthologs (minimize false positives, even at the expense of incurring false negatives) we chose for detailed further analysis the set of 145 genes that were differentially expressed in the alarm pheromone response and conserved in all the Eutheria (placental mammals) species (altogether 10 species in InParanoid, ranging from *B*.*taurus* to *H*. *sapiens*) considered in this study. The p-value for over-representation of orthologs of placental mammals in this set was actually smaller than 1e-6 (see Methods), which constitutes a correlation effectively impossible to have occurred by chance. Similarly, the most significantly conserved genes for all the insect species in the Old_vs_Young set were identified by a correlation effectively impossible to have occurred by chance (also with a p-value smaller than 1e-6). A larger set of genes (conserved in mouse and human but not necessarily in all 10 eutherian species) was also analyzed, as was a smaller set of genes conserved in all the vertebrates. Generally, the mouse-and-human set showed very similar GO enrichment patterns to the eutherian set, while the all-vertebrate set showed far fewer enriched ontology classes. Results of this analysis are provided in supplementary material.

In each of the three classes of bees (soldier, forager, guard) where we have both a CG gene set (differential gene expression between bees raised in predominantly African and European colonies) and a WG gene set (differential gene expression between genetically African and genetically European honey bee), we compared the degree of enrichment in orthologs with other metazoans. There was greater enrichment in orthologs in the CG set than in the WG set (p = .043 for guards, p < .0005 for foragers, p < .0005 for soldiers). The soldier cg-wg orthology is especially interesting for two reasons. Firstly the overall degree of orthology is much greater for the soldiers than for the foragers or guards. Secondly the most dramatic behavioral difference between the African and European bees is the behavior of the soldiers. The degree of difference between the soldier cg and wg orthologies is visualized in Fig 3F, which shows the cumulative fractional difference of the two distributions of p-values for pairwise orthology enrichment between the honey bee and the other 54 organisms represented in the analysis. It is important to note that the behavioral phenotype of the soldiers corresponds mainly to the phenotype of the colony in which they were raised. The cross-fostered soldiers are phenotypically much like the other soldiers in their colony, but differ in gene expression patterns. Our finding speaks to the general issue of the interaction between nature and nurture in defining social behavior, suggesting that if we wish to draw inferences for other metazoans from the different behavior of African and European honey bees, we must consider how the colony socializes individual bees. At the genomic level, this suggests that the overall genetic composition of African and European colonies (which would presumably be reflected in the nurturing environment in the hive, but is beyond the scope of the current study) is perhaps more important than the genetic differences of individual bees for understanding the broader comparative relevance of strain differences in behavior. Note also that the pattern of orthology enrichment across the metazoa is quite different for the soldier cg set than for the alarm pheromone set. Whereas the alarm pheromone set showed enriched orthology particularly for the highly social placental mammals, the orthology enrichment for the soldier cg set is higher for closer relatives to the honey bee, most notably the arthropods—most of whom are not eusocial. To summarize the orthology results:

- The alarm pheromone set showed a high degree of orthology with other metazoa and most especially with placental mammals, a higher degree of orthology than with the arthropods that are the nearer relatives to the honey bees.
- The “cg” sets, consisting of African bees reared in European hives and vice versa, showed a higher degree of orthology across the metazoan than the “wg” sets, consisting of bees raised in the hives of their own strain. The absolute degree of orthology was much greater for soldier sets than for either the forager or the guard sets. Unlike the alarm pheromone set, for the soldier cg set the highest degree of orthology was with the honey bee’s close relatives, the arthropods.

### Gene Ontology Analysis of Placental Mammal-Conserved Alarm_Pheromone Genes

**Fig 4:**
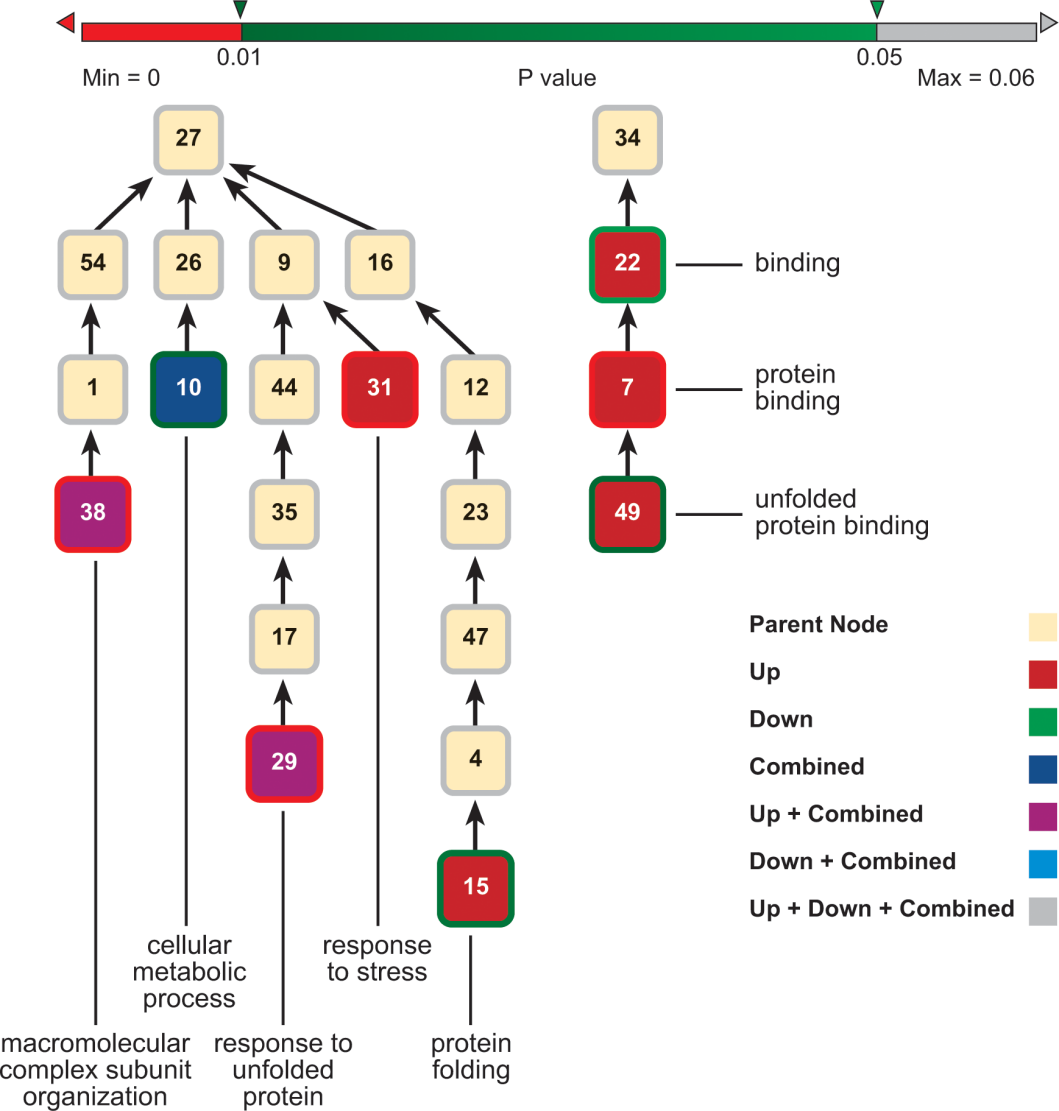
GO Biological Process and Molecular Function Trees (Benjamini-corrected p-value cutoff = 0.05) for the “Eutheria-conserved” Alarm_Pheromone Genes” Human Orthologs. This figure shows the Biological Process (left) and Molecular Function (right) Trees. “Eutheria-conserved” means that genes have orthologs in all eutherian species included in InParanoid database. Nodes with red bounds are GO terms with p-value (Benjamini p-value) < = 0.01. Nodes with various green bounds are nodes with p-value between 0.01 and 0.05. Nodes with white bounds (or no bounds as it is the same as the background color) are not themselves enriched but are parents of enriched terms in the GO hierarchy. Enriched nodes are labeled with the names of the GO term. All nodes are numerically tagged. The correlations between the numerical tags and the names of the GO categories is given in Table 3. Separate GO analysis are done for all the up-regulated “Eutheria-conserved” Alarm_Pheromone Genes” Human orthologs (Up”), all the down-regulated orthologs (“Down”), and all regardless of regulation (“Combined”). The results are indicated by the colors of the node faces as follows: GO category enriched in the up-regulated subset only is red; GO category enriched in the down-regulated subset only is green; GO category enriched in the complete set is deep blue; GO category enriched in the up-regulated subset and the complete set is purple; GO category enriched in the down-regulated subset and the complete set is light blue; GO category enriched in both subsets and in the total set is gray. For example, a Biological Process GO term indexed “29” and identified as “response to unfolded protein” is seen as significant in the analysis for up-regulated and for all genes (Hence it is in purple, the,”Down+Combined” color, and bordered in red because the p-value was below 0.01). All the names for the enriched GO terms and their parents are in Table 3 (The tables and this tree are designed to be complementary to each other. The trees show the overall architecture of the relationships among GO categories while the tables provide more detail) and in S2 Table. Corresponding information in spreadsheet form is provided for the “Mouse-and-human conserved” and “All-Vertebrate-Conserved” sets in S2 Table.

**Fig 5:**
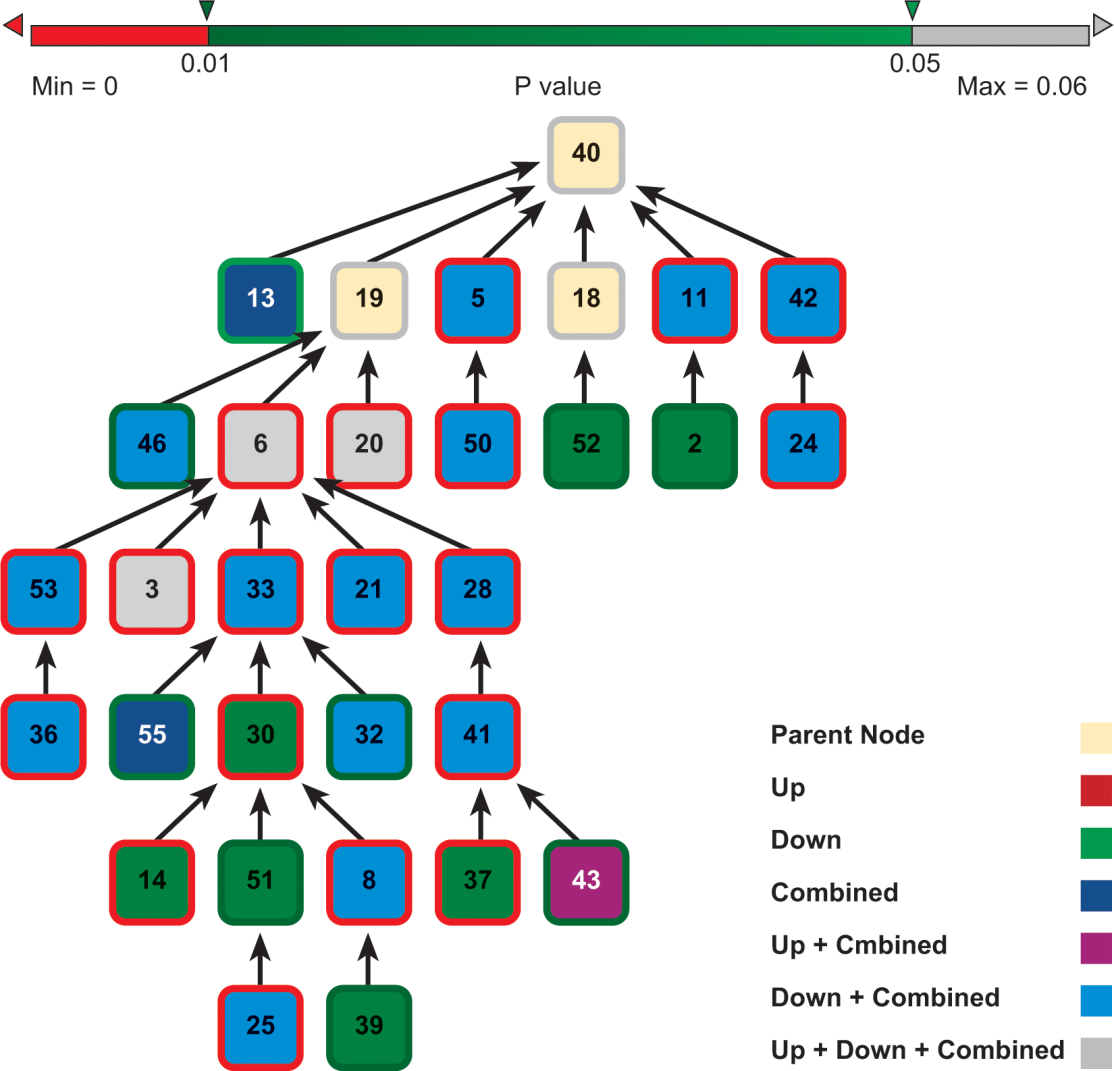
GO Cellular Component Trees (Benjamini-corrected p-value cutoff = 0.05) for the “Eutheria-conserved” Alarm_Pheromone Genes” Human Orthologs. This figure shows the Cellular Component tree. “Eutheria-conserved” defined as in Fig 4 legend. Nodes with red bounds are GO terms with p-value (Benjamini p-value) < = 0.01. Nodes with red bounds are GO terms with p-value < = 0.01. Nodes with various green bounds are nodes with p-value between 0.01 and 0.05. Nodes with white bounds (or no bounds as it is the same as the background color) are not themselves enriched but are parents of enriched terms in the GO hierarchy. Separate GO analysis are done for all the up-regulated “Vertebrate-conserved” Alarm_Pheromone Genes” Human orthologs (“Up”), all the down-regulated ones (“Down”), and all regardless of regulation (“Combined”). The results are indicated by the colors of the node faces as follows: GO category enriched in the up-regulated subset only is red; GO category enriched in the down-regulated subset only is green; GO category enriched in the complete set is deep blue; GO category enriched in the up-regulated subset and the complete set is purple; GO category enriched in the down-regulated subset and the complete set is light blue; GO category enriched in both subsets and in the total set is gray. The exact names for these indexed GO terms are in Table 4 and in S2 Table. Corresponding information about the “mouse-and-human-conserved” and “vertebrate-conserved” gene sets is found in S2 Table.

We used the DAVID suite of programs to identify Gene Ontology (GO) categories that were over-represented in the 145 alarm pheromone-responsive genes mentioned above, relative to their overall incidence in the human genome (131 of these 145 genes” human orthologs have Entrez annotations), at p-values of 0.01 and 0.05 (Benjamini-corrected for multiple hypothesis assumption). For better comparison, we performed three separate GO analyses: 1) for all these 131 genes, 2) for the 73 up-regulated genes 3) for the 58 down-regulated genes. The results are summarized in Figs 4 and 5 and in Tables 3 and 4. Tables 3 and 4 provide the names of the enriched GO categories, together with the p-value for their enrichment. The GO output analysis output, upon which Figs 4 and 5 and Tables 3 and 4 are based, is shown in spreadsheet form in S2 Table. The analysis below is based specifically on the gene set that was conserved among all the Eutheria. We also did the analysis on a larger set of genes conserved in the mouse and human but not necessarily in all the Eutheria. The results of that analysis was practically identical to the Eutheria-conserved set, so the verbal analysis below applies to that set as well.

**Table 3:** Exact GO term names for GO Trees for the ‘Eutheria-conserved’ Alarm_Pheromone Genes’ Human Orthologs(Part 1, Biological Process, Molecular Function).

Index	ID	Term	Pvalue	Parent	Children	Set Desc
27	GO:0008150	biological_process	>0.05		9,54,16,26	Parent Node
9	GO:0050896	response to stimulus	>0.05	27	44,31	Parent Node
44	GO:0042221	response to chemical stimulus	>0.05	9	35	Parent Node
35	GO:0010033	response to organic substance	>0.05	44	17	Parent Node
17	GO:0035966	response to topologically incorrect protein	>0.05	35	29	Parent Node
29	GO:0006986	response to unfolded protein**	5.63E-04	17		Up+Both
31	GO:0006950	response to stress**	0.00403	9		Up
54	GO:0071840	cellular component organization or biogenesis	>0.05	27	1	Parent Node
1	GO:0016043	cellular component organization	>0.05	54	38	Parent Node
38	GO:0043933	macromolecular complex subunit organization**	0.00957	1		Up+Both
16	GO:0008152	metabolic process	>0.05	27	12	Parent Node
12	GO:0071704	organic substance metabolic process	>0.05	16	23	Parent Node
23	GO:0043170	macromolecule metabolic process	>0.05	12	47	Parent Node
47	GO:0044260	cellular macromolecule metabolic process	>0.05	23	4	Parent Node
4	GO:0044267	cellular protein metabolic process	>0.05	47	15	Parent Node
15	GO:0006457	protein folding*	0.0215	4		Up
26	GO:0009987	cellular process	>0.05	27	10	Parent Node
10	GO:0044237	cellular metabolic process*	0.0159	26		Both
45	GO:0051789	response to protein stimulus**	0.00315	35		Up+Both
48	GO:0034621	cellular macromolecular complex subunit organization*	0.0221	38		Up+Both
34	GO:0003674	molecular_function	>0.05		22	Parent Node
22	GO:0005488	binding*	0.0438	34	7	Up
7	GO:0005515	protein binding**	0.00363	22	49	Up
49	GO:0051082	unfolded protein binding*	0.0151	7		Up

This table corresponds to the tree in Fig 4 and is designed to correspond to the topology of the tree. Thus entries in the table follow the vertical lineages in the tree starting with the left-most vertical lineage. “Index” column is the index number used in Fig 4. ‘Term’ column is marked according to p-value (Benjamini p-value, see Methods) significant level: p-values that are ≤ 0.01 are marked “**”, p-values that are between 0.01 and 0.05 are marked “*”. ‘Parent’ and ‘Children’ columns list the indexes of the parent/children node(s) of each node. ‘Set_Desc’ column delineates whether the enrichment is among the up-regulated, down-regulated, or up- and down-regulated components combined of the differentially regulated set. Terms of “Parent Node” are terms that are parent node of other enriched GO terms.

**Table 4:** Exact GO term names for GO Trees of the ‘Eutheria-conserved’ Alarm_Pheromone Genes’ Human Orthologs(Part 2, Cellular Component).

Index	ID	Term	Pvalue	Parent	Children	Set Desc
40	GO:0005575	cellular_component	>0.05		19,5,42,11,18,13	Parent Node
19	GO:0044464	cell part	>0.05	40	6,20,46	Parent Node
6	GO:0044424	intracellular part**	6.36E-06	19	28,3,53,33,21	Up+Down+Both
28	GO:0043229	intracellular organelle**	0.00104	6	41	Down+Both
41	GO:0043231	intracellular membrane-bounded organelle**	7.47E-04	28	43,37	Down+Both
43	GO:0005634	nucleus*	0.0140	41		Up+Both
37	GO:0005739	mitochondrion**	0.00325	41		Down
3	GO:0005737	cytoplasm**	4.65E-06	6		Up+Down+Both
53	GO:0044444	cytoplasmic part**	5.28E-04	6	36	Down+Both
36	GO:0005829	cytosol**	4.13E-04	53		Down+Both
33	GO:0044446	intracellular organelle part**	0.00726	6	30,32,55	Down+Both
30	GO:0044429	mitochondrial part**	0.00712	33	8,51,14	Down
8	GO:0044455	mitochondrial membrane part**	0.00216	30	39	Down+Both
39	GO:0005746	mitochondrial respiratory chain*	0.0111	8		Down
51	GO:0031966	mitochondrial membrane*	0.0145	30	25	Down
25	GO:0005743	mitochondrial inner membrane**	0.00677	51		Down+Both
14	GO:0005740	mitochondrial envelope*	0.0179	30		Down
32	GO:0031967	organelle envelope*	0.0129	33		Down+Both
55	GO:0044428	nuclear part*	0.0196	33		Both
21	GO:0019866	organelle inner membrane**	0.00727	6		Down+Both
20	GO:0005622	intracellular**	2.31E-05	19		Up+Down+Both
46	GO:0031975	envelope*	0.0122	19		Down+Both
5	GO:0032991	macromolecular complex**	3.12E-06	40	50	Down+Both
50	GO:0043234	protein complex**	2.06E-04	5		Down+Both
42	GO:0043226	organelle**	0.00101	40	24	Down+Both
24	GO:0043227	membrane-bounded organelle**	6.81E-04	42		Down+Both
11	GO:0044422	organelle part**	0.00741	40	2	Down+Both
2	GO:0031090	organelle membrane*	0.0177	11		Down
18	GO:0044425	membrane part	>0.05	40	52	Parent Node
52	GO:0070469	respiratory chain*	0.0157	18		Down
13	GO:0031974	membrane-enclosed lumen*	0.0458	40		Both

‘Index’ column is the index number used in the Fig 5. ‘Term’, ‘Parent’, ‘Children’ and ‘Set_Desc’ columns are defined as in Table 3.

Several of the same GO categories appeared in the results of more than one of the three analyses (up-regulated, down-regulated, all differentially expressed). Three GO categories were enriched in all three of the analyses, all three in the “Cellular Component” category (Table 4). They are: GO:0005737 (cytoplasm—“All of the contents of a cell excluding the plasma membrane and nucleus, but including other subcellular structures”), GO:0005622 (intracellular—“The living contents of a cell; the matter contained within (but not including) the plasma membrane, usually taken to exclude large vacuoles and masses of secretory or ingested material. In eukaryotes it includes the nucleus and cytoplasm.”), and GO:0044424 (intracellular part—essentially the same definition as GO:0005622 and with the same parent term, GO:0044464 (cell part). (Definitions in quotation marks are from EBI QuickGO[20]). The enrichment of these three terms in all of the three categories of differentially expressed genes means that few of the differentially expressed gene products reside in the plasma membrane, and both up-regulated and down-regulated genes were enriched for gene products found in other parts of the cell. The rest of the Cellular Component categories provided more specificity with respect to the locations of up-regulated and down-regulated genes.

Examination of enrichment in “Biological Processes” categories revealed several insights (Fig 4). There was a strongly enriched GO category under “cellular component organization or biogenesis” [node 54]—“macromolecular complex subunit organization” [node38] (Benjamini p-value = 0.0096). This enrichment suggests that the human pattern orthologous to the expression pattern of the honey bee alarm pheromone response involves protein complex organization and biogenesis. This GO term was not significantly enriched for down-regulated genes. 2) “Cellular metabolic process” [node10] was also an enriched GO term (Benjamini p-value = 0.016). This suggests that the human pattern orthologous to the expression pattern of the honey bee alarm pheromone response involves modulation of metabolism. 3) More specialized categories within the “response to stimulus” GO term were “response to stress” [node 31] and “response to unfolded protein” [node 29]. Taken together, these enrichments suggest that the human response pattern that is orthologous to the honey bee alarm pheromone response also involves responses to chemical and possibly other stimuli. It is plausible that the response to unfolded protein seen in this section of the tree was related to protein metabolism and biogenesis, and the protein complex assembly that was simultaneously being up-regulated during the overall organism response as indicated in other parts of the tree. “Protein folding” [node 15] was also enriched.

Gene Ontology analysis for molecular function revealed that that all the enriched GO terms fall under one general category—“binding” (22). GO analysis for cellular component (Fig 5 and Table 4) revealed the enrichment pattern included multiple cell components—cytoplasm, nucleus, mitochondria (mostly significant for down-regulated genes) and other organelles, protein, and possibly other macromolecular complexes. This was consistent with the biological processes and the molecular functions enriched in our analyses, which are localized in in a variety of cell components.

Since the members of the gene set from which these inferences are derived were conserved across the eutherians, it is plausible that the inferences are valid for eutherians in general. However, it should be reiterated that the results described in this section do not refer to the totality of either the honey bee alarm pheromone response or of a complete network in humans and other vertebrates. Rather, they refer to components of the honey bee alarm pheromone response network that are widely conserved in eutherians and have a well-defined GO classification in humans. These components were presumably present and possibly part of an interacting network in the last common ancestor of the human and the honey bee about 670 million years ago. Both the honey bee alarm pheromone network and networks in eutherians that share these components will undoubtedly have other different non-shared components particular to their respective classes of organism.

### Genes in the Set Associated with Neural Disease

Tables 5 and 6 show genes in our analysis set annotated with enriched GO biological process terms that have been implicated in neural and behavioral disorders, and those biological process terms with which they are associated that are also included in the list of enriched terms for the complete alarm pheromone set. This list was constructed by manual inspection of literature and OMIM databases, so is not comprehensive. The results of a GO analysis for this set of 25 genes is given in S4 Table, showing all biological process terms enriched to a p-value of 0.05 or better. The overwhelming majority of the enriched biological processes relate to metabolism in a way that would pertain to many different types of cells in addition to brain cells. Protein folding and organization of macromolecular complexes also appear as enriched categories. These genes are selected for both a specific brain response in the honey bee and also for broad conservation in the placental mammals. The interesting feature of this analysis is the convergence of three factors: 1) implication in human mental disease, 2) differential expression in the honeybee in response to a conspecific language element (the alarm pheromone) and 3) broad conservation across the placental mammals. It appears at least in part that several varieties of mental illness are based on issues related to evolutionarily deeply rooted and broadly conserved genes, as opposed to being solely related to genes specific to human cognition and behavior, or even specific to brain or neural function.

**Table 5:** Examples of Behavior/neural-related ‘Eutheria-conserved’ Alarm_Pheromone Genes’ Human Orthologs (Part 1).

Entrez ID	Name	GO Terms related	Neural Disease/behavior related
10963	STIP1	GO:0006950~response to stress	Aggressive Behavior(PMID: 21784300,association study)
			autism(PMID: 23838888)
7388	UQCRH	GO:0043933~macromolecular complex subunit organization,GO:0044237~cellular metabolic process	Alzheimer's disease(PMID: 14555243, indirectly related)
1103	CHAT	GO:0044237~cellular metabolic process	Alzheimer's disease(PMID: 15364407,PMID: 2166243,PMID: 16797789,PMID: 2054656)
			dementia(PMID: 10443555,PMID: 9143263)
522	ATP5J	GO:0044237~cellular metabolic process	Alzheimer's disease(PMID: 18332434,PMID: 22008262)
			autism(PMID: 17322880)
4116	MAGOH	GO:0044237~cellular metabolic process	anxiety behavior(PMID: 23638902,indirectly related—inferred by known dimerization with, and regulatory influence of,RBM8a)
3308	HSPA 4	GO:0006950~response to stress,GO:0006986~response to unfolded protein,GO:0034621~cellular macromolecular complex subunit organization,GO:0043933~macromolecular complex subunit organization,GO:0051789~response to protein stimulus	bipolar disorder(PMID: 19766166)
			Spinocerebellar Ataxia Type 7(PMID: 16039988)
			neurodegenerative diseases(PMID: 10562718,PMID: 11044589(A general reference to chaperones))
4716	NDUFB1	GO:0044237~cellular metabolic process	dementia(PMID: 20573273)
9801	MRPL19	GO:0044237~cellular metabolic process	dyslexia(PMID: 17309879,PMID: 21165691,PMID: 23209710)
4715	NDUFB9	GO:0044237~cellular metabolic process	fatal familial insomnia(PMID: 23430483)
			autism spectrum disorder(PMID: 24453408)
6434	TRA2B	GO:0044237~cellular metabolic process	frontotemporal dementia(PMID: 22456266,PMID: 23818142)
			fatal familial insomnia(PMID: 23430483)
8564	KMO	GO:0006950~response to stress,GO:0044237~cellular metabolic process	Huntington(PMID: 21640374,PMID: 20942784)
1410	CRYAB	GO:0006457~protein folding,GO:0006950~response to stress,GO:0043933~macromolecular complex subunit organization,GO:0044237~cellular metabolic process	leukodystrophy (PMID: 1407707,PMID: 1743282,PMID: 8393618)
			Picks disease(PMID: 1382240,PMID: 18091558,PMID: 7575218)
2288	FKBP4	GO:0006457~protein folding,GO:0034621~cellular macromolecular complex subunit organization,GO:0043933~macromolecular complex subunit organization,GO:0044237~cellular metabolic process	major depressive disorder(PMID: 19199039,PMID: 15565110,PMID: 19545546)

Based on the Gene Ontology result of Eutheria-conserved’ Alarm_Pheromone Genes’ Human Orthologs, genes related to the significant biological process GO terms(Benjamini p-value ≤ 0.05) are presented here.

**Table 6:** Examples of behavior/neural-related ‘Eutheria-conserved’ Alarm_Pheromone Genes’ Human Orthologs(Part 2).

Entrez ID	Name	GO Terms related	Neural Disease/behavior related
3312	HSPA8	GO:0006457~protein folding,GO:0006950~response to stress,GO:0006986~response to unfolded protein,GO:0044237~cellular metabolic process,GO:0051789~response to protein stimulus	neurocognitive and behavioral defects(PMID: 18855024,PMID: 21056629(behavioral study in mice, not directly causative))
84246	MED10	GO:0044237~cellular metabolic process	neurodegeneration(PMID: 18929508,indirectly related)
8879	SGPL1	GO:0044237~cellular metabolic process	neurodegeneration(PMID: 21331079)
645	BLVRB	GO:0044237~cellular metabolic process	neurodegeneration(PMID: 7682296,PMID: 8845972)
9374	PPT2	GO:0044237~cellular metabolic process	neuronal ceroid lipofuscinosis(PMID: 10051407)
5683	PSMA2*	GO:0044237~cellular metabolic process	Parkinson(PMID: 21069393,PMID: 15455214)
478	ATP1A3	GO:0044237~cellular metabolic process	parkinsonism(PMID: 17282997)
			Alzheimer's disease(PMID: 2035524)
			Depression(PMID: 18068248)
1460	CSNK2B	GO:0006950~response to stress,GO:0006986~response to unfolded protein,GO:0051789~response to protein stimulus	Parkinson's disease(GeneCard Inferred)
4709	NDUFB3	GO:0044237~cellular metabolic process	Parkinson's disease(PMID: 17211632)
3301	DNAJA1	GO:0006457~protein folding,GO:0006950~response to stress,GO:0006986~response to unfolded protein,GO:0044237~cellular metabolic process,GO:0051789~response to protein stimulus	Parkinson's disease(PMID: 22343013)
7334	UBE2N	GO:0006950~response to stress,GO:0044237~cellular metabolic process	schizophrenia(PMID: 12363385,PMID: 20109112)
11080	DNAJB4	GO:0006457~protein folding,GO:0006950~response to stress,GO:0006986~response to unfolded protein,GO:0044237~cellular metabolic process,GO:0051789~response to protein stimulus	stress-related(PMID: 24511526)

Based on the Gene Ontology result of Eutheria-conserved’ Alarm_Pheromone Genes’ Human Orthologs, genes related to the significant biological process GO terms(Benjamini p-value ≤ 0.05) are presented here.

## Discussion

This study was designed to examine the plausibility of the premise that the genomic networks underlying a response to a stimulus for social behavior (alarm pheromone response in honey bees) might have counterparts conserved in mammals, even though mammals do not use this particular alarm pheromone and the last common ancestor between honey bees and mammals lived approximately 670 million years ago [11]. Based on results from two different orthology databases, we found that the honey bee genes differentially expressed in response to alarm pheromone were more strongly conserved in orthologs to mammals than in orthologs to other metazoans, including those more closely related to the honey bee (nonsocial insects). We hypothesize that these orthologous sets are conserved remnants of a network responding to conspecific signals that first emerged in a common ancestor of insects and vertebrates and has been selectively conserved in social metazoa.

The reader will have noted that the experimental context of this paper was done on material from whole brains. For processing of conspecific signals such as spoken or written language in humans, many imaging studies show that several different regions of the brain are simultaneously activated. We therefore believe that whole brain studies such as ours are useful in revealing underlying commonalities of mechanism, but should be complemented by region-specific analyses.

It should be noted that this particular study deals only with those parts of the putative conserved network that are differentially expressed in response to the external signal. There may be other genes that are part of the network, but are present at relatively steady levels. This may be the reason for the conspicuous lack of genes for plasma membrane proteins in the “cellular component” category of enriched GO classes found in this study (Table 4). Plasma membrane proteins must be involved in any response to external signals, but their role in mediating between extracellular stimuli and intracellular response does not necessarily require either up- or down-regulation in immediate response to the alarm pheromone stimulus.

By contrast, genes in our study whose products reside in the nucleus were upregulated, genes in the mitochondria and other organelles were downregulated, and significant numbers of genes in the remainder of the cell were differentially regulated in both directions. Our results indicate that alarm pheromone exposure triggers significant physical remodeling of intracellular molecular signaling machinery. At the core of sociality is the ability to transmit and respond to complicated signals from conspecifics [21]. This is widely thought to involve the ability of nervous systems to rapidly increase the activity of some cellular networks and reduce the activity of others in response to these signals [22]. Our results suggest that there is another level of complexity enabled by the ability to remodel macromolecular interaction networks within cells in response to a transient signal from conspecifics, such as alarm pheromone. This remodeling would allow for changes in responses to subsequent signals, i.e., for stimuli experienced presently to enable individuals to “predict” the future. Since our results are based on enrichment of orthologous genes between honey bees and mammals, this hypothesis implies the original development of this remodeling ability in an ancient common ancestor of mammals and insects.

Based on these results we offer the following speculation about possible mechanisms for macromolecular remodeling within brain cells and organismic sociality. The time scales for protein folding, for binding reactions, and for assembly of macromolecular complexes from pre-existing elements, can be fractions of a second, so these processes can take place rapidly enough to be consistent with the time scale of the alarm pheromone response. However, transcription and translation of genes will take many seconds or minutes [23]. The necessarily faster time scale for the alarm pheromone response suggests involvement of a more rapid remodeling process, perhaps involving microRNAs, which have for several years been postulated to play a role in synaptic plasticity [24]. The recently developed CLIP-seq technology [25] permits comprehensive identification of microRNA binding sites in a variety of tissues, including the brain [26]. Thus it should be possible in the future to explore this speculation and experimentally characterize the roles of specific microRNA in brain remodeling in response to conspecific signals.

Perhaps one aspect of the dichotomy between highly social and solitary animals is in the ability of the individual brain cells in social animals to remodel their interaction networks in response to signals from conspecifics. This ability would not come without a tradeoff, since maximal speed of response would be achieved by activating existing hard-wired networks. Thus evolutionary niches have persisted for both highly social and less social animals, with less social animals optimized for speed of response to all stimuli by activating hard-wired circuits, while highly social animals have developed the ability to remodel molecular circuits in response to signals from conspecifics—a process which results in necessarily slower response. This may also apply to the evolution of the most complex form of conspecific communication–human language. In this view the corresponding circuits underlying honey bee chemical language and human auditory language would be “phenologs”; that is, varying phenomes based on orthologous genes [27].

## Methods

### Statistical Analyses of Ortholog Gene Counts

The p-values in Table 2 for the average number of metazoan orthologs for each data set were computed as follows: For each experimental data set, random sets of matching size were sampled from the 7462 honey bee genes that were present in InParanoid database [18] and spotted on the array, and the average number of orthologs per gene was calculated for each random set. This random sampling was repeated one million times and the number of random sets with average ortholog number equal to or larger than the experimental set was counted. The count divided by 10^6^ gave us the p-value for the average ortholog number of the test set. S1 and S2 Figs show how the p-values of the average ortholog number of Forager_CG and Alarm_Pheromone sets were calculated. The p-values for the total number of orthologs of each set for each species (Fig 3) were computed similarly.

For calculating the p-value for the CG-WG difference, the KS-test p-values for the CG-WG difference for Soldier, Forager and Guard (0.026, .122, and .612 respectively) were combined using Fisher’s method [28].

For calculating the p-value for over-representation of orthologs of placental mammals in the Alarm_Pheromone set and over-representation of orthologs of insects in the Old_vs_Young set, p-values in each species (S1 Table) were also combined using Fisher’s method.

In presenting and discussing the results, we use the term “conserved” to be measured by the number of orthologs that a particular sequence has; i.e., the more orthologs a gene or protein has in other species, the more “conserved” the gene is.

### Gene Ontology Analysis

Enrichment of the conserved gene sets in particular Gene Ontology categories was determined using the functional annotation tool in the Database for Annotation, Visualization, and Integrated Discovery (DAVID)[29]. All parameters are default except that we use GO_TERM_*_ALL instead of GO_*_FAT. Extra functional analyses (of various qualities) were also included: OMIM_Disease [30], COG_Ontology [31], SP_PIR_Keywords [32], Up_Seq_Feature [33], BBID [34], BioCarta [35], Kegg_Pathway [36], Interpro Domains [37], Pir_Superfamily [38], and Smart [39].

The raw Gene Ontology results of “Eutheria-conserved”,Alarm_Pheromone genes are listed in S2 Table. Figures of Gene Ontology trees (Figs 4 and 5) were generated by Python scripts and Cytoscape [40]. Benjamini-Hochberg corrected p-values provided by DAVID are used for indication of significance [29].

Scientific references about the relationship between behavior/neural functions and genes associated with significant GO terms were identified with GeneCard and manual search with Google Scholar, using keywords “behavior”,”disease”,”neural”, and”aggression”.

### Identifying Metazoan Orthologs of Honey Bee Genes

First, honey bee genes that showed up on the microarray studied in Alaux et al [15] were selected. This was done based on the annotation file of the Honey Bee Oligonucleotide Microarray [15]. Out of many available methods [14] of defining orthologs, two were chosen, InParanoid [18] and OrthoMCL [41]. InParanoid has the most extensive coverage of the honey bee proteome and other proteomes of completed genomes in searchable ortholog databases. Out of all these “microarray-present” honey bee genes, we identified those that are also present in InParanoid. This was done by mapping the BeeBase IDs (which are the IDs used in the data set from Alaux et al [15]) to NCBI Refseq IDs (which are the IDs used in InParanoid for honey bee). 7462 of these “microarray-present” honey bee genes are present in InParanoid. At the time of the analysis, there were 100 eukaryotic species in InParanoid with 54 of them (including *Apis mellifera*) being metazoan species. With *S*. *cerevisiae* added as a control, the data set used for our analysis had 55 species, which we interrogated for orthology with the 7462 InParanoid honey bee proteins.

## Supporting Information

S1 Narrative S1 and S2 Figs illustrate the computational method for computing the p-value for enrichment of individual datasets of honey bee genes in orthologs contained in other metazoan. Randomly selected sets of honey bee genes of the same size as the experimental set were repeatedly examined for orthologs in the other species. The distribution is shown as a normal distribution peaking at approximately 32. The different distribution widths in S1 and S2 arise from the different numbers of genes in the corresponding experimental sets. S1 Fig illustrates the results for a particular experimental set that not enriched, and indeed probably relatively depleted in orthologs, since the genes in the set average only about 30 orthologs. The p-value (probability of achieving the experimental ortholog number by chance) is given by dividing the area contained in the red part of the distribution by the total area under the distribution (red plus green). S2 Fig is the corresponding figure for the alarm pheromone set that is analyzed intensively in this paper, which is seen to be strongly enriched in orthologs to other species. S1 Table is the spreadsheet providing the raw numbers underlying Fig 3A and 3B. S2 Table is the spreadsheet providing the raw numbers underlying Figs 4 and 5, and also corresponding numbers for two other data sets. One is the alarm pheromone set with orthologs in mouse and human, but not necessarily in all the placental mammals. This is a larger set of genes than the one described in more detail in the main body of the paper. The patterns of gene ontology enrichment are almost identical to those of the data set described in the main body of this paper. The other additional set is the alarm pheromone set with orthologs in all the vertebrate species. This is a smaller set of genes, and contains very few enriched gene ontology categories. S3 Table provides in spreadsheet form a comprehensive tabulation of the individual honey bee gene and orthology identifications used in this study. S4 Table shows the gene ontology enrichment categories of the set of genes identified in Tables 5 and 6 of the main text.
(DOCX)

S1 Fig Distribution of average ortholog number for Forager_CG set. This distribution was generated by random sampling, one million times. Each time, a random set of the same size of Forager_CG set was retrieved from InParanoid’s whole honey bee gene population (those that were on the microarray, as defined in Methods). The Average Ortholog Number of the random set was then calculated as follows: [total number of orthologs of all genes within the set]/[number of genes within the set].
(TIF)

S2 Fig Distribution of average ortholog number for Alarm_Pheromone set. This distribution was generated in the same was as in S1 Fig, using the Alarm Pheromone set.
(TIF)

S1 Table Table S1. P-values for the number of total orthologs in each species for each set. Raw data for Fig 3; p-values were calculated by random sampling, from one million sets of randomly chosen genes, each set having the same number of genes as the corresponding experimental set.
(XLSX)

S2 Table Spreadsheet showing all computed Gene Ontology enrichment results summarized in Figs 3 and 4 and Tables 4 and 5 and also corresponding results for the mouse-and-human set and the all-vertebrate set. (XLSX)

S3 Table Spreadsheet showing differential expression and orthology patterns that provided the basis for this study. (XLSX)

S4 Table Gene Ontology analysis of the 25 human orthologs to Alarm Pheromone genes identified as associated with mental illness. (PNG)
