# Supplementary material for: Conservation in Mammals of Genes Associated with Aggression-Related Behavioral Phenotypes in Honey Bees
Source: PLoS Comput Biol. 2016 Jun 30;12(6):e1004921. doi: 10.1371/journal.pcbi.1004921 (PMC4928799; doi:10.1371/journal.pcbi.1004921)
Supplement: S1 Narrative — Randomly selected sets of honey bee genes of the same size as the experimental set were repeatedly examined for orthologs in the other species. The distribution is shown as a normal distribution peaking at approximately 32. The different distribution widths in S1 and S2 arise from the different numbers of genes in the corresponding experimental sets. S1 Fig illustrates the results for a particular experimental set that not enriched, and indeed probably relatively depleted in orthologs, since the genes in the set average only about 30 orthologs. The p-value (probability of achieving the experimental ortholog number by chance) is given by dividing the area contained in the red part of the distribution by the total area under the distribution (red plus green). S2 Fig is the corresponding figure for the alarm pheromone set that is analyzed intensively in this paper, which is seen to be strongly enriched in orthologs to other species. S1 Table is the spreadsheet providing the raw numbers underlying Fig 3A and 3B. S2 Table is the spreadsheet providing the raw numbers underlying Figs 4 and 5, and also corresponding numbers for two other data sets. One is the alarm pheromone set with orthologs in mouse and human, but not necessarily in all the placental mammals. This is a larger set of genes than the one described in more detail in the main body of the paper. The patterns of gene ontology enrichment are almost identical to those of the data set described in the main body of this paper. The other additional set is the alarm pheromone set with orthologs in all the vertebrate species. This is a smaller set of genes, and contains very few enriched gene ontology categories. S3 Table provides in spreadsheet form a comprehensive tabulation of the individual honey bee gene and orthology identifications used in this study. S4 Table shows the gene ontology enrichment categories of the set of genes identified in Tables 5 and 6 of the main text. (DOCX) [file pcbi.1004921.s001.docx]

Supplementary Narrative.

Figures S1 and S2 illustrate the computational method for computing the p-value for enrichment of individual datasets of honey bee genes in orthologs contained in other metazoan. Randomly selected sets of honey bee genes of the same size as the experimental set were repeatedly examined for orthologs in the other species. The distribution is shown as a normal distribution peaking at approximately 32. The different distribution widths in S1 and S2 arise from the different numbers of genes in the corresponding experimental sets. Figure S1 illustrates the results for a particular experimental set that not enriched, and indeed probably relatively depleted in orthologs, since the genes in the set average only about 30 orthologs. The p-value (probability of achieving the experimental ortholog number by chance) is given by dividing the area contained in the red part of the distribution by the total area under the distribution (red plus green). Figure S2 is the corresponding figure for the alarm pheromone set that is analyzed intensively in this paper, which is seen to be strongly enriched in orthologs to other species.

Table S1 is the spreadsheet providing the raw numbers underlying Figures 3a and 3b.

Table S2 is the spreadsheet providing the raw numbers underlying Figures 4 and 5, and also corresponding numbers for two other data sets. One is the alarm pheromone set with orthologs in mouse and human, but not necessarily in all the placental mammals. This is a larger set of genes than the one described in more detail in the main body of the paper. The patterns of gene ontology enrichment are almost identical to those of the data set described in the main body of this paper. The other additional set is the alarm pheromone set with orthologs in all the vertebrate species. This is a smaller set of genes, and contains very few enriched gene ontology categories.

Table S3 provides in spreadsheet form a comprehensive tabulation of the individual honey bee gene and orthology identifications used in this study.

Table S4 shows the gene ontology enrichment categories of the set of genes identified in Tables 5 and 6 of the main text.
