# Supplementary figures and images for: Conservation in Mammals of Genes Associated with Aggression-Related Behavioral Phenotypes in Honey Bees
Source: PLoS Comput Biol. 2016 Jun 30;12(6):e1004921. doi: 10.1371/journal.pcbi.1004921 (PMC4928799; doi:10.1371/journal.pcbi.1004921)

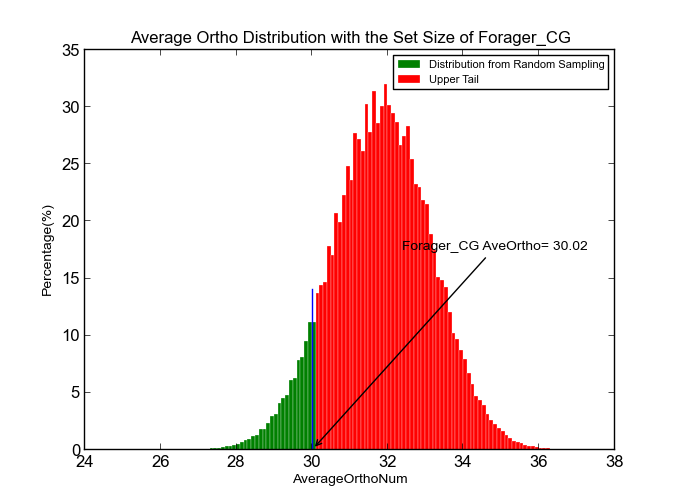

Supplement: S1 Fig — This distribution was generated by random sampling, one million times. Each time, a random set of the same size of Forager_CG set was retrieved from InParanoid’s whole honey bee gene population (those that were on the microarray, as defined in Methods). The Average Ortholog Number of the random set was then calculated as follows: [total number of orthologs of all genes within the set]/[number of genes within the set]. (TIF) [file pcbi.1004921.s002.tif]

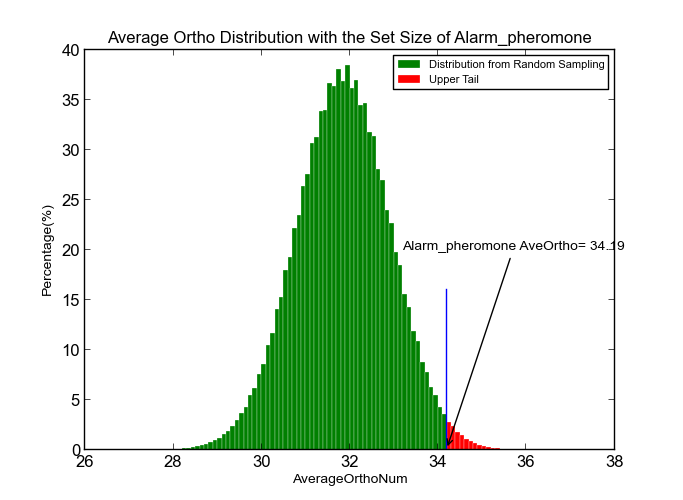

Supplement: S2 Fig — This distribution was generated in the same was as in S1 Fig, using the Alarm Pheromone set. (TIF) [file pcbi.1004921.s003.tif]

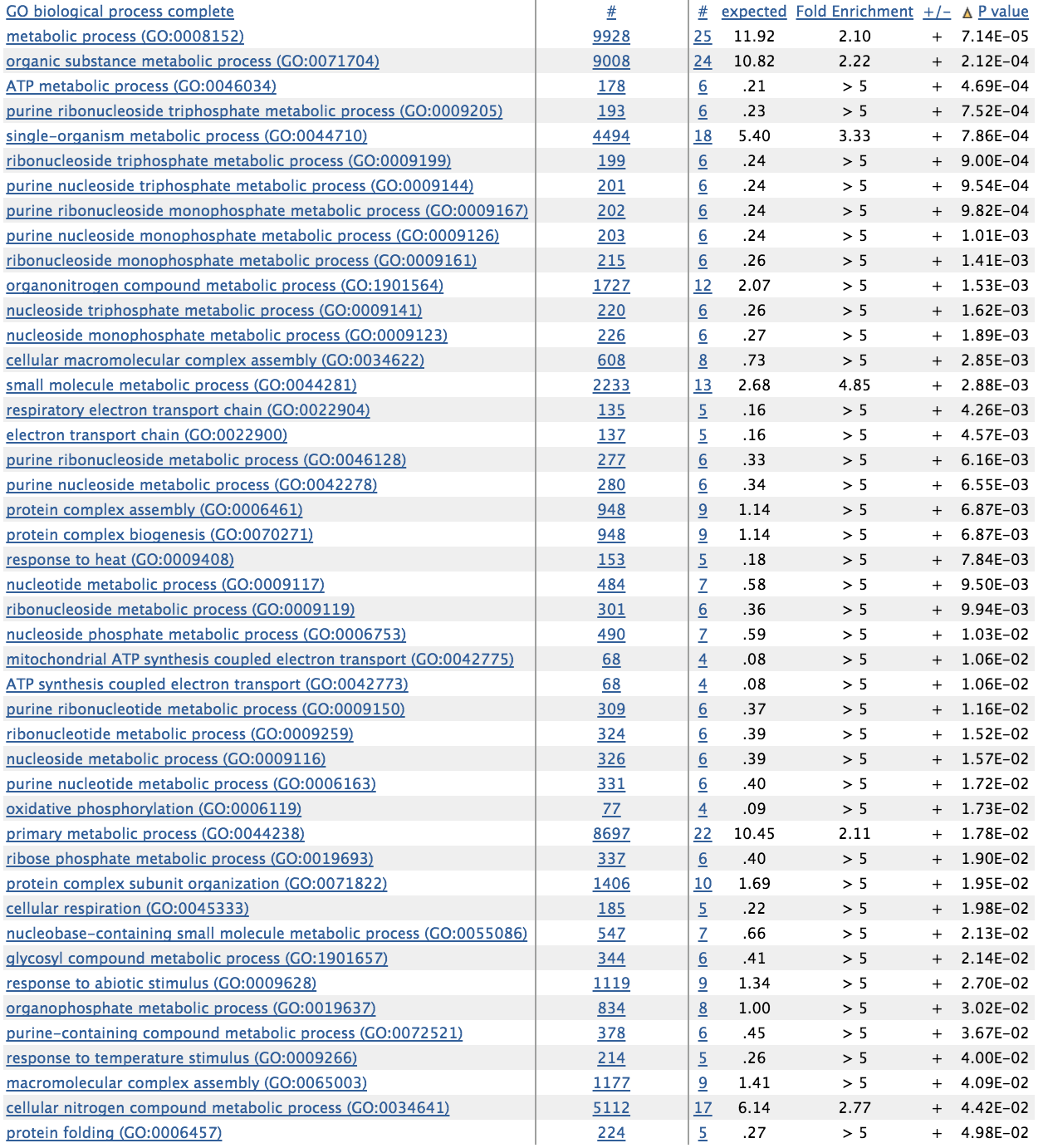

Supplement: S4 Table — (PNG) [file pcbi.1004921.s007.png]
